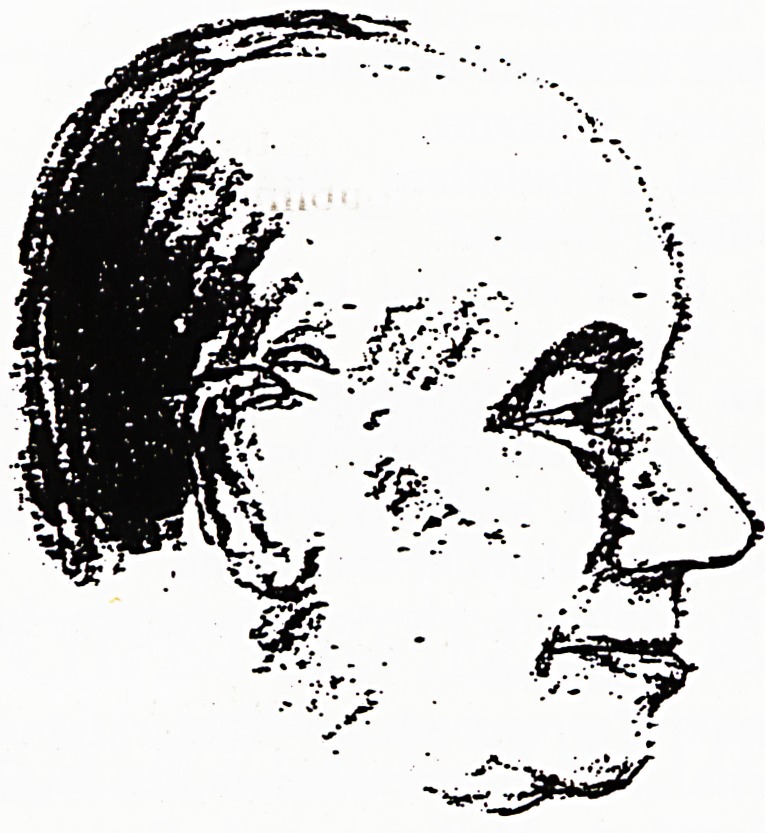# Introduction

**Published:** 1988-11

**Authors:** C. B. Perry

**Affiliations:** Emeritus Professor of Medicine, University of Bristol


### 250 Years of Patient Care in the Bristol Royal Infirmary

### INTRODUCTION

Although the Hospital Management had a celebration to mark the 250th Anniversary of the Foundation of the Bristol Royal Infirmary, the medical staff felt a need for a special occasion to commemmorate the achievements of our great teaching hospital. Professor Alan Read takes the credit for the initial ideas which eventuated in the meeting recorded in this issue.

The Postgraduate and Education committee coordinated the academic and social proceedings and Dr David Wilkins lent his invaluable expertise to ensuring that the audio-visual facilities ran to perfection. He also recorded the proceedings on tape and passed these to the editor to assist him in making this account.

Attending the symposium was Professor John Emery, Professor of Paediatrics in the University of Sheffield. He was noticed during the proceedings to be making sketches of the speakers and was persuaded to allow them to be used to illustrate our account. In his letter accompanying his drawings he says "I don't think these sketches have any particular merit, but I send them to you to do with as you will". All the sketches were interesting as character studies and about half of them were thought to have caught an excellent likeness and are reproduced below. Inevitably they have suffered somewhat in reproduction and reduction in size.

A final thanks go to Mrs Penny Lee, our Postgraduate Secretary, whose tireless efforts ensured that the plan fell perfectly into place. It was an exhilarating day, particularly for the remarkable glimpses of the future that we were given by our speakers.
Cameron Kennedy.
Chairman of the Postgraduate Committee

### A meeting to commemorate the 250th anniversary of the first intake of patients to the Infirmary

### The Bristol Royal Infirmary

**Professor C.B.Perry**
Emeritus Professor of Medicine, University of Bristol.

250 years ago tomorrow the Infirmary admitted its first inpatients, 17 men and 17 women to the original building in Lower Maudlin Lane. By the end of the century this had been entirely rebuilt, the West wing though completed in 1798 for financial reasons was not put into commision until 1810. In the mid-19th century another storey was added and the Infirmary then looked very much as the old building looks today. In 1910 the King Edward Memorial building on the other side of the road was added and recently the architectural monstrosity which now disfigures Maudlin St. Today we should remember those who made the buildings possible. Those who started the whole movement were stimulated they said by 'desiring, as far as in them lies, to find some remedy for the great misery of our poor neighbours'. First of these were John Bonython, a Cornishman who became the first physician and John Elbridge who was the Controller of Customs, born in Massachusetts, he at his own expense built and equipped one ward and when he died left £5,000 to the institution in his will, a very great deal of money at the beginning of the 18th century.

Until 1948 the institution was entirely supported by voluntary contributions, subscriptions and legacies, its history chequered by a series of financial crises, always surmounted by a special appeal largely initiated by one man, or by a large legacy which suddenly fell in. The greatest of these crises was probably in 1904 when with a debt of £15,000 the President and Treasurer, Sir Charles Cave resigned and said the only thing to do was to close 6 wards and sell all investments until the debt was cleared. Instead, the committee in their wisdom invited George White, founder of the Bristol Aereoplane Company, to succeed him, he accepted, saying it was a challenge which no real Bristolian could refuse. He launched an appeal which he and his brother largely financed, cleared off the debt, refurbished the gloomy old building and built the new one, the Edward 7th Memorial, opened in 1910. At the end of this the debt was £12,000, and the annual income was greatly exceeded by the expenditure. Then in 1916 a legacy from a Mr Capern of £45,000 saved the situation. In 1920 Mr Harry Wills put into trust securities to the value of £110,000 on the condition that the BRI and the General Hospital would amalgamate by the end of the year, the Infirmary, pretty hard up again, agreed; the General Hospital for the moment flush, refused. and so the Infirmary had the whole £110,000.

The reputation of a hospital is not made by buildings but by those who work in them and we should remember some of these today. Edward Long Fox senior, physician 1786-1816, became a great authority on lunacy and was called to Windsor in consultation on George III. Richard Smith junior, surgeon 1796-1843, collected every scrap of information there was about the Infirmary before his time and his papers are an invaluable record not only of the Infirmary but of the City. William Budd, physician 1847-1862, the father of Epidemiology, long before any bacteria had been described he realised that typhoid was spread by water and speculated that pulmonary tuberculosis was infectious, a thought that came to him 'unbidden, so to speak, when I was walking alone on observatory hill one evening'. John Beddoe, physician 1847-1862, according to Sir Arthur Keith the greatest anthropologist of the Victorian era. Greig-Smith, surgeon 1899- 1907, who died tragically young at the age of 47, he wrote a text book of surgery which ran into four editions and was translated into French. Finally, Munro-Smith, surgeon 1897-1909, wrote the monumental history of the BRI without which this introduction would not have been possible.

## Figures and Tables

**Figure f1:**